# Double jeopardy--drug and sex risks among Russian women who inject drugs: initial feasibility and efficacy results of a small randomized controlled trial

**DOI:** 10.1186/1747-597X-7-1

**Published:** 2012-01-10

**Authors:** Wendee M Wechsberg, Evgeny Krupitsky, Tatiana Romanova, Edwin Zvartau, Tracy L Kline, Felicia A Browne, Rachel Middlesteadt Ellerson, Georgiy Bobashev, William A Zule, Hendrée E Jones

**Affiliations:** 1 RTI International, Research Triangle Park, North Carolina, USA; 2 Valdman Institute of Pharmacology, Pavlov State Medical University, St. Petersburg, Russia

**Keywords:** HIV, women, female IDUs, Woman-Focused intervention, Russia

## Abstract

**Background:**

With HIV prevalence estimated at 20% among female injecting drug users (IDUs) in St. Petersburg, Russia, there is a critical need to address the HIV risks of this at-risk population. This study characterized HIV risks associated with injecting drug use and sex behaviors and assessed the initial feasibility and efficacy of an adapted Woman-Focused intervention, the Women's CoOp, relative to a Nutrition control to reduce HIV risk behaviors among female IDUs in an inpatient detoxification drug treatment setting.

**Method:**

Women (*N *= 100) were randomized into one of two one-hour long intervention conditions--the Woman-Focused intervention (*n *= 51) or a time and attention-matched Nutrition control condition (*n *= 49).

**Results:**

The results showed that 57% of the participants had been told that they were HIV-positive. At 3-month follow-up, both groups showed reduced levels of injecting frequency. However, participants in the Woman-Focused intervention reported, on average, a lower frequency of partner impairment at last sex act and a lower average number of unprotected vaginal sex acts with their main sex partner than the Nutrition condition.

**Conclusion:**

The findings suggest that improvements in sexual risk reduction are possible for these at-risk women and that more comprehensive treatment is needed to address HIV and drug risks in this vulnerable population.

## Background

Russia is an emerging epicenter of the global HIV epidemic [1], accounting for 66% of all newly registered HIV cases in Eastern Europe and Eurasia [2]. The geographical nexus of Russia's HIV epidemic is St. Petersburg, with an HIV prevalence rate of 30-47% among injecting drug users (IDUs) [3]. Additionally, 80-90% of the HIV cases in St. Petersburg are associated with IDUs, many of whom are unaware of their HIV status [4-6]. Further, HIV morbidity is reported to be highest among IDUs in St. Petersburg [7].

In earlier studies, Russian women in general and female IDUs in particular appeared to be at high risk of HIV, but HIV prevalence among them was relatively low [8,9]. However, between 1996 and 2006, the number of HIV-infected women increased rapidly from 29% to 44% [2,9]. In St. Petersburg, HIV prevalence among female IDUs was estimated to be 20% [10]. Consequently, there is a critical need to address the HIV risks of female IDUs [11-13].

Because of the multifaceted risks women face, they are at high risk for contracting and spreading HIV. For example, sharing contaminated injecting equipment and sexual transmission are the main causes of HIV infection for female IDUs [2,11,14]. Also, the power and control differential that female IDUs often experience in heterosexual relationships heightens their risk of HIV infection because out of fear of abuse by their sex partner and/or the need to obtain daily necessities for survival they may not be able to negotiate safer sex practices [15].

Research suggests a high correlation between sex risk behaviors and drug use [14,16]. Among IDUs, for example, drug use may impair judgment and increase risky sex behaviors, including unprotected sex, having multiple sex partners, or exchanging sex for drugs and everyday necessities [17]. In addition, injecting drug use has been reported to be a major factor in the increase of commercial sex workers in Russia [11]. Studies show that 15-50% of female IDUs in Russia engage in commercial sex work for money and/or drugs [8,11,18], yet some women also trade sex only for drugs. Evidence suggests that sexually active female IDUs, whether commercial sex workers or not, are a most-at-risk population in St. Petersburg and urgently need HIV prevention interventions.

Consequently, this study aimed (1) to characterize the risks for HIV associated with injecting drug use and sex behaviors among female IDUs in drug treatment in St. Petersburg, and (2) to assess the initial feasibility and efficacy of an adapted Woman-Focused intervention, the Women's CoOp [19], in reducing HIV risk behaviors. The Women's CoOp is considered a best-evidence intervention [20] and is based on empowerment theory [21] and principles of social cognitive theory [22]. It focuses on increasing education and information about substances to reduce use and gain personal power, enhancing skills and self-efficacy of implementing condom skills, and increasing assertiveness for protection within relationships and sexual negotiations [23].

## Methods

### Adapting the Women's CoOp Intervention and Procedures

The formative research stage of this study used in-depth interviews to examine the risk factors of Russian female IDUs and to adapt the Women's CoOp intervention. Participants in the in-depth interviews included female IDUs in the Leningrad Regional Center of Addictions (LRCA), an inpatient substance abuse treatment center. The interviews focused on learning more about drug use and risk, understanding sexual risk, and informing the adaptation of the Women's CoOp intervention to the Russian context [24].

The adaptation process involved modifying the original Women's CoOp to address injecting risks, adapting an attention control Nutrition intervention to fit Russian cultural food norms [25], making sure both interventions had equal one-hour sessions (two total sessions for each intervention), and translating both interventions into Russian [26]. Also, the data collection instrument used across the Women's CoOp studies needed measures to be inserted for injecting risk and to be translated and pretested. Human subject approvals were received in both the United States and Russia at each stage of this project. This approval was in compliance with the Helsinki Declaration and the final study approval was given by RTI International's Office of Research Protection and Ethics, Protocol Number 11870.

Some of the formative activities to support the adaptation provided a better understanding of the risk behaviors among these women and how to address these behaviors within the life context of Russian women. The results supported the notion of the intersection of disempowered women using drugs and facing increased HIV risk associated with their use of injecting equipment and high-risk sex practices. The most salient finding was that in order to maintain their own drug addiction, many boyfriends and husbands were sending the women out to trade sex for heroin.

### Treatment Setting

Typically, LRCA patients receiving treatment for alcohol and/or other drug abuse remain in the hospital for 3 to 4 weeks for treatment of withdrawal. Patients are stabilized during the first 7 to 10 days, and once stabilized they are detoxified. HIV testing also occurs during this period. In addition, related psychiatric and somatic problems are addressed during the inpatient stay.

The most common drugs used to treat the signs and symptoms of heroin withdrawal were clonidine, non-opioid analgesics, hypnotics, and an antidiarrheal. Patients do not receive any medication-assisted therapy, as it is illegal in Russia to use opioid agonist medications to treat addiction or pain. Substance abuse treatment also includes individual and group cognitive and behavioral relapse prevention therapy.

### Screening, Recruitment, and Randomization

Participants for the small randomized controlled trial (RCT) were drawn from inpatients at LRCA who were identified and approached by staff or from self-referrals based on posted advertisements within the clinic. Participation in the study was voluntary, and decisions to participate or not to participate did not affect receipt of treatment services. Eligibility criteria included being female, self-reported injection drug use in the past year, current LRCA substance abuse treatment for more than 4 days (total treatment varies from 7 days to 4 weeks), being 18 to 30 years old, being able to sign informed consent, not currently in any other research study, and providing verifiable locator information in the St. Petersburg area to facilitate contact by field staff for follow-up assessment. Women received an HIV antibody test at admission and were being detoxified, so no further drug testing was conducted. Women who met study eligibility criteria and who agreed to participate were read the informed consent document and provided written informed consent. The informed consent document included a description of the study and the randomization process. A total of 100 women were randomized into one of two 1-hour long intervention conditions--the Woman-Focused intervention (*n *= 51) or a Nutrition time-and-attention-matched control condition (*n *= 49).

### Data Collection and Study Process

All consents, data collection, and interventions were translated into Russian and back-translated by LRCA staff. Interviews were conducted in a private office by a trained female interviewer either immediately after or up to a few days post-consent. The intake instrument included questions on demographics, lifetime/current alcohol and other drug use, drug injecting practices, sex behaviors, conflict and victimization, mental health, and physical health. Because it was voluntary, after completion of the baseline interview, all participants were asked to return within the next few days for their first intervention appointment. A follow-up questionnaire was administered approximately 3 months post randomization.

Participants in both intervention conditions were asked to return in a week for the second intervention session. Although they were still inpatients, these requests were all voluntary and separate from patients' daily routines. Both intervention sessions lasted about 1 hour and were conducted by an LRCA staff psychologist. Upon completion of either intervention condition, women received 500 rubles (about USD$20) for their time. At the follow-up visit, which occurred about 3 months after the first intervention session, participants were asked to provide a urine sample for drug testing and took part in a single assessment interview comprising questions similar to the baseline questionnaire. This visit lasted about 90 minutes. At the completion of the follow-up appointment, the women received 1000 rubles (about USD$40) for their time, a completion certificate, and a risk-reduction kit.

### Woman-Focused Intervention

The Woman-Focused intervention was developed originally for substance-abusing African American women in the United States [19] and subsequently adapted for South African women [23,24]. This intervention is interactive, manual-driven, and focuses on empowering women through educational activities, skill-building demonstrations, guided practice, and role-playing. Information is presented on cue-cards that provide structure while allowing the interventionist the flexibility to tailor the content to the needs of individual participants. The sessions are delivered by a female staff psychologist who has been trained on the intervention.

The intervention adapted for Russian women comprises two sessions that address the risks women face in relation to their injection drug use and sex-risk behavior for HIV. Issues of violence among Russia women, including developing skills for violence prevention, are also discussed. Session 1 covers general information on HIV, risks associated with alcohol and other drug use, injection drug use, benefits of substance abuse treatment, hepatitis C virus, sexually transmitted infections, strategies for reducing sex risk, and harm reduction. Session 2 covers substance abuse and relationships, physical and sexual abuse, rape and violence prevention, ways of discussing and negotiating safer sex, HIV test results, and developing an action plan for reducing substance abuse and HIV risk.

Women randomized to the Woman-Focused condition received the intervention via a one-on-one session while they were LRCA inpatients. At the end of the second session, women created a personalized risk-reduction plan to help them reduce their sex risk behaviors, substance use, and physical and sexual victimization. Other LRCA staff conducted data collection, quality assurance, and data entry.

### Nutrition Intervention

The equal-attention control group received a two-session intervention adapted from the Colorado State University Extension Nutrition Program's "Eat Well for Less" (EWFL) curriculum. EWFL was developed with funding from federal and local food stamp programs and the US Department of Agriculture. It was chosen as the equal-attention control group for its reliance on best nutrition practices as well as its explicit discussion of healthy eating with limited resources [25].

EWFL is interactive in ways similar to the Woman-Focused intervention in that it encourages evaluating the pros and cons associated with healthful choices and guides the participant into goal-setting activities. It was adapted to make it more culturally appropriate for Russian women, including how staples in the Russian diet, such as the potato, can be cooked in several healthy ways. This adaptation also incorporated cabbage and other food groups, and demonstrated new ways to cook to retain the nutrients in foods as well as additional suggestions for a healthy lifestyle.

### Training and Supervision of Staff

With the assistance of a translator, the Principal Investigator helped to train the interventionists. The field supervisor trained the data collectors. A multilingual project director from LRCA served as a senior team member and conducted quality assurance and observations.

### Measures

Study interviews were conducted using a Russian-adapted Revised Risk Behavior Assessment (RRBA) for women [27]. Drug use measures (lifetime, past year, and past 30 days before treatment entry), assessed 10 specific substances: alcohol, marijuana, ecstasy, crack cocaine, cocaine, heroin, amphetamine/methamphetamine, tobacco, methadone, and "Jeff" or "ephedrone" (i.e., an oxidation product of ephedrine). The primary drug-related outcomes for this study were injection-related HIV-risk behaviors (i.e., receptive syringe sharing, indirect sharing [sharing of cookers and cottons], and syringe-mediated drug sharing [sharing liquefied drugs that have been prepared with water added from a used syringe]).

The RRBA contains questions about vaginal, oral and anal contact, which generate counts of these specific sex behaviors in the month prior to the interview, as well as the number of episodes where condoms are used. These items were computed to yield 30-day counts of unprotected vaginal intercourse, oral sex and anal intercourse, which represent the primary sex behavior outcomes of this study. "Impaired sex" was defined as having sexual relations under the influence of substances. This report focuses on drug and sex risk as the two primary outcomes.

### Follow-up Rates

At 3 months, 91% of the sample had completed interviews. Women were tracked for follow-up in the surrounding region.

### Analysis

Descriptive statistics were compiled on baseline characteristics of the participants. Using t-tests for continuous variables and chi-square tests of association for categorical variables, baseline differences were examined in key study outcomes by intervention condition to assess the equivalence of the study intervention conditions. Additionally, the results of t-tests and chi-square tests of association were examined at 3-month follow-up to determine if any post-intervention differences existed between participants in the Nutrition and Woman-Focused conditions.

Because the counts of risky behaviors were relatively high, ordinary regression analysis was used rather than Poisson. A general linear model was used for continuous outcomes, which included the baseline status and the intervention group indicator. Logistic regression using the same model was used for binary outcomes. Categorical variables were dichotomized. All of the analyses were conducted using SAS version 9.2. These analyses focused on drug use and sex risk and change at 3 months.

## Results

### Demographic information

Table 1 presents the participants' demographics as well as baseline information on key risk behaviors. Participants were, on average, 26 years old; the majority had at least a high school education (80%); and they were involved with a heterosexual main partner (68%). All of the participants were IDUs, with a mean age of 18.5 at the first time they injected drugs. Almost a quarter of the participants had an intimate partner help them the first time they injected drugs and 50% had shared works in their lifetime. More than half of the participants (57%) had been told that they were HIV-positive, as they were tested upon inpatient treatment entry. Slightly less than half of the sample had ever traded sex for drugs, money, clothing, shelter, or any other goods (44%).

**Table 1:** Study Sample (*N *= 100) Demographic Characteristics

	Total Sample (*N *= 100)	Nutrition Condition (*n *= 49)	Woman-Focused Intervention Condition (*n *= 51)
	**n (%)^a ^or *M *(*SD*)**		

Mean age	25.9 (SD = 3.0)	26.0 (SD = 2.8)	25.7 (SD = 3.1)
Education			
Less than high school	20 (20%)	12 (24.5%)	8 (15.7%)
High school or greater	80 (80%)	37 (75.5%)	43 (84.3%)
Marital status			
Single	17 (17%)	8 (16.3%)	9 (17.7%)
Involved, Married, or Living as Married	68 (68%)	32 (65.3%)	36 (70.6%)
Separated, Divorced, Widowed	15 (15%)	9 (18.4%)	6 (11.8%)
Ever told HIV+ (yes)	57 (57%)	26 (53.1%)	31 (60.8%)
Ever traded sex for drugs, money, clothing, shelter, or any other goods (yes)	44 (44.4%)	22 (44.9%)	22 (44.0%)
Ever used a female condom with main sex partner (yes)^b^	2 (2.8%)	1 (2.9%)	1 (2.7%)
Mean age at first time injected	18.5 (3.2 SD)	18.9 (3.3 SD)	18.1 (3.0 SD)
Intimate partner helped inject first time (yes)	22 (22%)	10 (20.8%)	12 (23.5%)
Ever shared works that had been used by someone else (yes)	50 (50%)	21 (42.9%)	29 (56.9%)

^a ^Percentage of participants who responded Yes within the given intervention group

^b ^Of those that had main sex partners

### Baseline risk behavior

Among the participants, 37% used protection the last time they had sex with their main partner, and they reported an average of eight unprotected vaginal sex acts with their main partner in the 30 days prior to entering treatment. Almost a third (30%) of the participants traded sex in the 30 days prior to entering treatment and they reported an average of four unprotected sex acts with sex-trading partners during that time. Almost 90% of the participants were impaired (i.e., used alcohol or other drugs) just before or during the last time they had sex, in contrast with 36% of their partners. In addition, 39% of the participants had unprotected vaginal sex while they were impaired.

Injection risk behaviors were prevalent in this sample, with all women injecting drugs; the most common drug being heroin. On average, women were injecting heroin almost daily prior to entering LRCA for drug abuse treatment, and admission to treatment was prior to enrollment in the study.

While all women in the study were heroin users, in the 30 days prior to entering treatment, 46% of the women also had injected cocaine and 44% also had injected "speedball" (i.e., a mixture of heroin and cocaine). In addition, participants engaged in risky drug use behaviors such as indirect sharing practices (i.e., sharing cotton, cooker/spoon, or rinse water) or syringe-mediated drug sharing (i.e., preparing drugs with water from a used syringe and/or measuring and dividing liquefied drugs with a used syringe--see Table 2). Other drugs used in the 30 days prior to entering treatment included marijuana (59%), ecstasy (53%), crack (46%), heroin (not injected, 52%), and "Jeff" (24%). However, because all of the study participants were in treatment for and were recently detoxified from heroin, the change in heroin injection behavior is the most salient drug outcome in the current study and therefore has the greater analytic focus on days of use.

**Table 2:** Baseline and 3-Month Follow-up Sex- and Drug-Risk Behaviors

	Baseline (N = 100)			3-Month Follow-up (N = 91)		
**Outcome Measure** **n (%)**^**a **^**or *M *(*SD*)**	**Baseline Total Sample** **(*N *= 100)**	**Nutrition Condition** **(*n *= 49)**	**Woman-Focused Intervention Condition** **(*n *= 51)**^**b**^	**Follow-up Total Sample** **(*N *= 91)**	**Nutrition Condition** **(*n *= 42)**	**Woman-Focused Intervention Condition** **(*n *= 49)**

Traded sex for drugs, money, clothing, shelter, or any other goods in 90 days prior to drug treatment (yes)	30 (30%)	16 (32.7%)	14 (27.5%)	38 (41.8%)	14 (33.3%)	24 (49.0%)
Protected last sex acts with main sex partner (yes)^c^	18 (36.7%)	9 (37.5%)	9 (36%)	47 (59.5%)++	16 (47.1%)	31 (68.9%)*
Mean number unprotected vaginal sex acts with main sex partner in 30 days prior to entering drug treatment^c^	7.8 (8.8 SD)	6.4 (7.8 SD)	9.0 (9.5 SD)	4.3 (5.8 SD)++	6.4 (6.8 SD)	2.6 (4.2 SD)**
Mean number unprotected sex acts with sex trading partner in 30 days prior to entering drug treatment	4.1 (19.6 SD)	7.5 (26.6 SD)	0.2 (0.6 SD)	2.3 (13.1 SD)	5.5 (20.6 SD)	0.1 (0.4 SD)
**Impaired last sex (yes)**						
Participant	87 (89.7%)	40 (85.1%)	47 (94.0%)	68 (78.2%)+	30 (75.0%)	38 (80.9%)
Partner	35 (36.1%)	15 (31.9%)	20 (40.0%)	20 (23.0%)	12 (30.0%)	8 (17.0%)
Impaired last vaginal sex - Unprotected (yes)	38 (39.2%)	18 (38.3%)	20 (40.0%)	26 (29.9%)	12 (30.0%)	14 (29.8%)
Last sex with main sex partner using female condom (yes)^c^	0	0	0	8 (12.1%)	0	8 (18.6%)*
Mean number of injections in past 30 days prior to drug treatment	96.7 (40.0 SD)	99.1 (41.6 SD)	94.6 (39.0 SD)	42.0 (40.2 SD)	44.6 (40.1 SD)	39.6 (40.6 SD)
Mean number of times used cotton, cooker/spoon, or rinse water used by someone else in past 30 days	8.0 (19.8 SD)	3.4 (6.8 SD)	12.0 (24.0 SD)	0.6 (2.7 SD)	1.2 (3.9 SD)	0.1 (0.4 SD)
Mean number of times someone else used your cotton, cooker/spoon, or rinse water in past 30 days	6.0 (17.8 SD)	3.1 (8.8 SD)	8.5 (22.7 SD)	1.2 (2.8 SD)	1.5 (3.1 SD)	1.0 (2.7 SD)
Mean number of times injected drugs mixed with water from someone else's syringe in the past 30 days (i.e. syringe-mediated drug sharing)	7.2 (18.3 SD)	1.7 (4.5 SD)	12.8 (24.5 SD)*	0.5 (1.6 SD)	0.9 (2.2 SD)	0.1 (0.5 SD)*
Mean days injected heroin only	28.5 (5.8 SD)	28.1 (6.9 SD)	28.9 (4.8 SD)	14.7 (13.0 SD)	15.4 (13.8 SD)	14.0 (12.3 SD)
**Any drugs injected**						
Cocaine only	41 (45.6%)	19 (44.2%)	22 (46.8%)	1 (1.1%)	1 (2.4%)	0
Heroin and cocaine	41 (44.1%)	19 (41.3%)	22 (46.8%)	3 (3.3%)	2 (4.8%)	1 (2.0%)
**Any drugs smoked/snorted**,						
Marijuana	43 (58.9%)	19 (55.9%)	24 (61.5%)	10 (11.0%)	4 (9.5%)	6 (12.2%)
Ecstasy only	43 (53.1%)	19 (51.4%)	24 (54.6%)	8 (8.8%)	3 (7.1%)	5 (10.2%)
Crack/cocaine only	41 (46.1%)	19 (44.2%)	22 (47.8%)	4 (4.4%)	1 (2.4%)	3 (6.1%)
Heroin only	42 (51.9%)	19 (48.7%)	23 (54.8%)	0	0	0
Jeff	19 (24.1%)	8 (21.1%)	11 (26.8%)	2 (2.2%)	1 (2.4%)	1 (2.0%)

^a ^Percentage of participants who responded Yes within the given intervention group, some variable have missing responses due to lack of relevance for individual participants.

^b ^"*" Markings of significance indicate significant group differences between the Nutrition and the Woman-focused intervention groups within time (i.e., baseline **or**3-month follow-up). "+"Markings of significance indicate significant over time differences either overall, or within the Nutrition or the Woman-focused intervention group.

^c ^Of those that had main sex partners and had intercourse with them.

*p < .05; **p < .01; ***p < .001

+p < .05; ++p < .01; +++p < .001

### Analysis over time

Analyses over time (mean or percentage presented in Table 2), paired t-tests (t) for continuous variables, and the McNemar test for the significance of change (S) for binary variables showed significant changes in several outcome variables. Sex-risk behavior frequencies showed some reduction over time. Primarily, there was a significant increase in the percentage of participants who used protection during the last sex contact with their main partner (Baseline = 36.7%; Follow-up = 59.5%; S = 7.12 [1], p = 0.01). Additionally, unprotected vaginal sex acts with main sex partner significantly reduced over time (t = 2.57 [50], p = 0.01) and this finding was driven by significant reductions in the Woman-Focused condition (t = 3.31 [27], p = 0.003) as compared with the Nutrition condition. Participants' impairment at last sex contact with any partner also showed significant improvement, with 89.7% of participants reporting some alcohol and/or other drug use at baseline before or during sex, whereas 78.2% of participants reported sexual contact while impaired at follow-up (S = 4.67 [1], p < .05).

The frequency of risky drug use injection behaviors, such as the average number of injections in the past 30 days, showed a significant reduction (Baseline *M *= 96.7; Follow-up *M *= 42.0; t = 10.35 [72], p < 0.0001). In addition, the average number of days that participants injected heroin by itself also showed a significant reduction (Baseline *M *= 28.5; Follow-up *M *= 14.7; t = 9.48 [89], p < 0.0001). Other past 30-day injection risk behaviors, such as the average number of times a participant injected drugs mixed with water from another injector's syringe, also showed a significant reduction (Baseline *M *= 7.2; Follow-up *M *= 0.5; t = 2.31 [28], p = 0.03). Finally, the number of times a participant shared their own cotton, cooker, or water showed a significant reduction (Baseline *M *= 6.0; Follow-up *M *= 1.5; t = 2.45 [71], p = 0.02) as well as the number of times a participant used a cotton, cooker, or water from someone else (Baseline *M *= 8.0; Follow-up *M *= 1.2; t = 2.32 [27], p = 0.03).

### Intervention effect

To estimate the effect of the intervention on salient outcomes, the analysis dataset was limited to only those participants who completed the baseline and follow-up interviews. An assessment of attrition cases yielded no significant differences on baseline characteristics between participants who completed the study and those who dropped out. Subsequently, a series of regression models was conducted for each of the outcomes (sex- and injection-risk behaviors) and the value of the intervention coefficient and the effect of the baseline value were estimated. The results of these analyses (regression coefficients with their 95% confidence intervals [CIs]) are presented in Table 3.

**Table 3:** Regression Analyses With 3-Month Follow-up Behaviors as Outcomes and Controlling for Each Outcome Variable's Baseline Status*

	Regression coefficient corresponding to the Baseline Status **Parameter Estimate (SE)**^**a**^ **/Odds Ratio (95% CI)**^**b**^	Regression coefficient corresponding to the Intervention Group Parameter Estimate (SE) /Odds Ratio (95% CI)
	**Parameter Estimate (*SE*) or Odds Ratio (95% *CI*)**	

Traded sex for drugs, money, clothing, shelter, or any other goods in past 90 days (yes)	1.02 (0.27-3.84) Wald Χ^2 ^= 0.001 (1), p = 0.98	0.92 (0.25-3.34) Wald Χ^2 ^= 0.02 (1), p = 0.90
Any protected last sex act with main sex partner (yes)	0.23 (0.05-1.00) Wald Χ^2 ^= 3.87 (1), p = 0.05	1.66 (0.46-6.03) Wald Χ^2 ^= 0.60 (1), p = 0.44
Mean number of unprotected vaginal sex acts with main sex partner in past 30 days	0.24 (0.09) t = 2.69 (1), p = 0.01	-4.56 (1.64) t = -2.77 (1), p = 0.008
Mean number of unprotected sex acts with sex- trading partner in past 30 days	-0.07 (0.25) t = -0.27 (1), p = 0.79	-9.18 (14.58) t = -0.63 (1), p = 0.54
Impaired last sex (yes)		
Participant	0.18 (0.04-0.78) Wald Χ^2 ^= 5.26 (1), p = 0.02	1.43 (0.47-4.29) Wald Χ^2 ^= 0.40 (1), p = 0.53
Partner	0.17 (0.05-0.54) Wald Χ^2 ^= 9.13 (1), p = 0.003	0.31 (0.10-0.98) Wald Χ^2 ^= 3.97 (1), p = 0.05
Impaired last unprotected vaginal intercourse (yes)	0.02 (0.004-0.07) Wald Χ^2 ^= 30.75 (1), p < 0.0001	0.91 (0.22-3.83) Wald Χ^2 ^= 0.02 (1), p = 0.90
Mean number of injections in past 30 days	0.25 (0.12) t = 2.07 (1), p = 0.04	-3.90 (9.26) t = -0.42 (1), p = 0.67
Mean number of times used cotton, cooker/spoon, or rinse water used by someone else in past 30 days	0.01 (0.02) t = 0.57 (1), p = 0.57	-1.16 (0.83) t = -1.39 (1), p = 0.18
Mean number of times someone else used your cotton, cooker/spoon, or rinse water in past 30 days	0.01 (0.02) t = 0.36 (1), p = 0.72	-0.56 (0.69) t = -0.81 (1), p = 0.42
Mean number of times injected drugs mixed with water from someone else's syringe in past 30 days (i.e. syringe-mediated drug sharing)	0.01 (0.01) t = 0.48 (1), p = 0.63	-0.76 (0.47) t = -1.61 (1), p = 0.12
Mean number of days injected heroin in past 30 days	0.21 (0.24) t = 0.88 (1), p = 0.38	-1.27 (2.76) t = -0.46 (1), p = 0.65

*The regression has the form: Outcome = Intercept + b1* Baseline Status+ b2* Intervention Group + error or Logit[P(Outcome = 1)] = Intercept + b1* Baseline Status+ b2* Intervention Group

^a ^Table input for linear regression ^b ^Table input for logistic regression

As indicated previously, intervention status had a significant effect on the average number of unprotected vaginal sex acts with a main partner, with participants in the Woman-Focused condition reporting significantly fewer unprotected sex acts than participants in the Nutrition condition. Intervention status also had a significant effect on participants reporting partner impairment at last sex contact with their main partner, with fewer participants in the Woman-Focused condition reporting partner impairment than participants in the Nutrition condition.

## Discussion

Several notable findings emerged from this first examination of the feasibility and efficacy of this evidence-based intervention modified for female IDUs in St. Petersburg. The double jeopardy of women's drug risk and sex risk makes this study vital to addressing these issues. First, the fact that almost 6 of every 10 participants (57%) were HIV-positive is highly disproportional and emphasizes the need to implement effective HIV prevention and intervention for this high-risk population of women. Living within a male-dominated society that often leaves them without other economic opportunities and unable to negotiate safer sex behaviors, these women and the men they have sex with represent important drivers of the HIV epidemic in Russia. Second, the fact that, on average, participants in each intervention condition reduced drug injection in the number of days used and number of times used in a month affirms the importance of substance abuse treatment to improve drug risk outcomes. Third, the results suggest that, on average, the Woman-Focused condition produced greater reductions in sex-risk behaviors (e.g., less unprotected vaginal sex acts) with main sex partners, as compared with the Nutrition condition. These results are similar to the results seen with the Women's CoOp in other cultural settings [28].

Taken together, the results from this initial feasibility study of the adaption of the Women's CoOp for female IDUs in Russia suggest that this intervention was successful in teaching women the needed skills to negotiate condom use and protected sex with main partners. However, although the intervention did not stop drug use at 3 months or trading behaviors that are also associated with how drugs are obtained, harm-reduction methods were utilized in teaching about drug use and injecting risk, and how to reduce risk. The findings are not definitive, but they underscore the importance of future research to more fully examine the efficacy of the Women's CoOp with female IDUs in Russia.

Although significant reductions in both drug use and sex-risk behaviors occurred in both intervention conditions, no difference is evident between the conditions with regard to reductions in heroin use. This may have been confounded by the fact that all of the study participants were in treatment for and recently detoxified from heroin, and some contamination between the conditions may have occurred. Russian law allows treatment of opioid dependence with antagonist medications such as naltrexone, which is superior to placebo [29]; however, the study participants were in detoxification and did not receive it. Furthermore, Russian law does not allow for treatment with opioid agonist medications, such as methadone or buprenorphine, which are superior to placebo conditions and yield improvement in long-term outcomes [30,31]. Access to the full continuum of medication options for opioid dependence would likely improve the transient benefits of any short-term brief intervention with detoxification. These fleeting benefits are evidenced by the fact that, as seen in the average number of days heroin was injected, at their 3-month follow-up interview most participants were injecting heroin every other day.

The strengths of this initial feasibility and efficacy study include its design features, such as randomization to conditions and that the control condition was matched for time and attention. Additionally, the 91% completion rate at the 3-month follow-up interview leaves little opportunity for missing data to compromise the results.

However, the study has several limitations. First, the extent to which small intervention studies, such as this one, successfully conducted within a hospital setting can be generalized to larger community settings is unknown. Second, in contrast to some other examinations of the Women's CoOp, this study tested the feasibility of the intervention in the context of inpatient substance abuse treatment. This setting and the services received in this context may have compromised the ability to see improvements specific to the Women's CoOp where contamination may have occurred. Third, the small sample size may have provided insufficient power to detect significant changes in some of the outcomes or any long-term sustainability, especially with regard to sex-risk reduction. However, with the piloted sample, the study had 76% power to detect an observed effect size of 0.5 at a probability level of 0.05. Fourth, despite the RRBA being translated and revised for this study, further research establishing its reliability and validity with Russian female IDUs is needed. Nevertheless, the results from this study suggest that future research should examine the Women's CoOp as a potentially efficacious intervention for a population in dire need of such an HIV prevention intervention. In addition, this study used a nutrition intervention for comparison, which does not provide information on the relative efficacy of the intervention compared with other HIV prevention interventions. Finally, given the large number of tests and a liberal alpha level of 0.05, there is a likelihood of Type I error in these findings.

Overall, this small trial was successful for the Woman-Focused condition (e.g., addressing drug risk and sex risk). Larger trials with female IDUs across settings are needed to replicate and extend the promising results found here. In addition, policy changes are needed within the Russian substance abuse treatment system to legalize a continuum of proven treatment options for female IDUs who also are at risk for HIV, other health related issues, and gender-based violence. Finally, because most of the women in this at-risk population are of childbearing age, successful interventions for them have the potential to also benefit future generations.

## Competing interests

The authors declare that they have no competing interests.

## Authors' contributions

WW was the Principal Investigator and conceptual developer and writer of this manuscript; EK was the Co-Principal Investigator and was responsible for data oversight and quality assurance and contributed to the writing; TR provided and supervised the behavioral intervention; EZ was the project director and responsible for administrative and organizational issues; TK conducted the data analyses; FB assisted in the literature review and contributed to the writing; RE conducted the field training and quality assurance and data edits for this manuscript; GB was the senior statistical analyst; WZ contributed to the analyses and writing; HJ contributed to the writing of this manuscript.
